# Adjusted mortality of extracorporeal membrane oxygenation for acute myocardial infarction patients in cardiogenic shock

**DOI:** 10.1097/MD.0000000000033221

**Published:** 2023-03-17

**Authors:** Jeong Cheon Choe, Sun-Hack Lee, Jin Hee Ahn, Hye Won Lee, Jun-Hyok Oh, Jung Hyun Choi, Han Cheol Lee, Kwang Soo Cha, Myung Ho Jeong, Dominick J Angiolillo, Jin Sup Park

**Affiliations:** a Department of Cardiology and Medical Research Institute, Pusan National University Hospital, Busan, Korea; b Division of Cardiology, Jeonnam National University Hospital, Gwangju, Korea; c Division of Cardiology, University of Florida College of Medicine, Jacksonville, FL.

**Keywords:** cardiogenic shock, extracorporeal membrane oxygenation, myocardial infarction, survival

## Abstract

Cardiogenic shock (CS) is a common cause of death following acute myocardial infarction (MI). This study aimed to evaluate the adjusted mortality of venoarterial extracorporeal membrane oxygenation (VA-ECMO) with intra-aortic balloon counterpulsation (IABP) for patients with MI-CS. We included 300 MI patients selected from a multinational registry and categorized into VA-ECMO + IABP (N = 39) and no VA-ECMO (medical management ± IABP) (N = 261) groups. Both groups’ 30-day and 1-year mortality were compared using the weighted Kaplan–Meier, propensity score, and inverse probability of treatment weighting methods. Adjusted incidences of 30-day (VA-ECMO + IABP vs No VA-ECMO, 77.7% vs 50.7; *P* = .083) and 1-year mortality (92.3% vs 84.8%; *P* = .223) along with propensity-adjusted and inverse probability of treatment weighting models in 30-day (hazard ratio [HR], 1.57; 95% confidence interval [CI], 0.92–2.77; *P* = .346 and HR, 1.44; 95% CI, 0.42–3.17; *P* = .452, respectively) and 1-year mortality (HR, 1.56; 95% CI, 0.95–2.56; *P* = .076 and HR, 1.33; 95% CI, 0.57–3.06; *P* = .51, respectively) did not differ between the groups. However, better survival benefit 30 days post-ECMO could be supposed (31.6% vs 83.4%; *P* = .022). Therefore, patients with MI-CS treated with IABP with additional VA-ECMO and those not supported with ECMO have comparable overall 30-day and 1-year mortality risks. However, VA-ECMO-supported survivors might have better long-term clinical outcomes.

## 1. Introduction

Cardiogenic shock (CS) is reported to be the most common cause of death following acute myocardial infarction (MI).^[1]^ Fluid administration for the maintenance of volume status is the first-line therapy for CS. In addition, inotropes and vasopressors are used to improve the risk of cardiac mortality in over 90% of CS cases.^[2]^ In the Should We Emergently Revascularize Occluded Coronaries for Cardiogenic Shock (SHOCK) trial, early revascularization with initial medical stabilization yielded favorable long-term clinical outcomes.^[3]^ Regarding percutaneous coronary intervention (PCI) strategies for CS, current guidelines recommend culprit-only PCI followed by staged PCI after hemodynamic stabilization based on the results of the randomized, multicenter Culprit-Lesion-Only PCI versus Multi-vessel PCI in Cardiogenic Shock trial.^[4]^

There have been recent advances in the evidence-based management of patients in CS. However, CS’s 40 to 50% mortality rate has not changed in a decade.^[5]^ Mechanical circulatory support (MCS) reduces the need for inotropes and vasopressors and improves hemodynamic instability which could lead to unfavorable clinical outcomes.^[6]^ However, the intra-aortic balloon counterpulsation (IABP)-SHOCK II trial^[7]^ showed that the IABP does not provide survival benefits. This finding may lead to a decrease in the use of IABP and an increase in the use of other MCS methods, such as the use of percutaneous ventricular assist devices or extracorporeal membrane oxygenation (ECMO).^[8]^ However, despite the increase in the number of devices used for mechanical support in CS, data from randomized clinical trials regarding their effectiveness are lacking.

A small randomized controlled trial demonstrated no differences in cardiac function (50.0% vs 50.8%; *P* = .86) and 30-day mortality (19% vs 33%; *P* = .37) between using ECMO alone and medical management.^[9]^ Studies regarding the comparisons between the use of ECMO and IABP separately in MI-CS revealed a significantly increased risk of 30-day mortality in the ECMO group.^[10–13]^ One study compared the mortality between ECMO ± IABP and IABP alone (81% vs 74%; *P* = .5),^[14]^ while another revealed a lower incidence of IABP ± ECMO compared with IABP alone (33% vs 68%; *P* = .01).^[15]^ However, these observational registry-based studies only reported unadjusted incidences of short-term mortality, which could lead to false interpretations of the effect of ECMO support in MI-CS.

Sustaining adequate perfusion and unloading of the ventricle during CS is essential to improve clinical outcomes. Venoarterial ECMO (VA-ECMO) can provide >4.5 L of oxygen per minute without the need for surgery. In addition, oxygenation is generally preferred over other types of MCS.^[16]^ Moreover, a recent systematic analysis of 17 observational studies (3997 patients) concluded that ECMO support in addition to left ventricular (LV) unloading is associated with decreased 30-day mortality risk (relative ratio, 0.79; 95% confidence interval [CI], 0.72–0.87; *P* < .00001).^[17]^ Therefore, VA-ECMO with unloading, in addition to early revascularization, could effectively maintain adequate perfusion and result in a good clinical outcome of MI-CS. This study aimed to evaluate the adjusted incidences of 30-day and 1-year mortality of ECMO + IABP over medical management ± IABP for acute MI patients with CS who underwent early revascularization.

## 2. Method

### 2.1. Study design and population

The study was conducted using data extracted from the Korea Acute Myocardial Infarction-National Institute of Health (KAMIR-NIH) registry from October 2011 to December 2015. The KAMIR-NIH is a prospective, multicenter registry study designed to characterize the clinical features and prognoses of acute MI in Korean patients. Data coordinators met with the study investigators at least twice a year to discuss the research and examine the study protocol.^[18]^

Patients were included in the study in accordance with the following inclusion criteria: age > 18 years; initial presentation with MI, which was diagnosed based on clinical manifestations; elevated levels of myocardial biomarkers, including creatinine kinase (CK)-myoglobin and troponin-I or T; and changes in the 12 leads of an electrocardiogram. CS was defined as a systolic blood pressure < 90 mm Hg for at least 30 minutes that required the administration of catecholamines, such as adrenaline, to maintain it at ≥90 mm Hg.^[19]^ In addition to this definition, at least one of the following pieces of evidence of end-organ damage was expected: altered mental status, cold skin and extremities, and decreased urine output (<30 mL/h). Furthermore, the included patients must have undergone early PCI. Conversely, patients who met at least one of the following criteria were excluded: coronary angiogram did not show a coronary artery disease; did not undergo PCI; underwent coronary artery bypass grafting; lost to follow-up; incomplete hemodynamic data; and did not initially present with CS. Only patients with MI complicated by CS were included. The included patients were categorized into VA-ECMO with IABP and no VA-ECMO support groups.

PCIs were performed according to the current practice guidelines. Any type of stent could be used without restriction. The decision to perform multi-vessel revascularization was left to the attending physician. The indications for VA-ECMO initiation were generally based on the Extracorporeal Life Support Organization guideline for Venoarterial Extracorporeal Membrane Oxygenation in Adult Cardiac Patients^[19]^; however, the final decision was at the discretion of the attending physician.

### 2.2. Clinical outcomes and statistical analysis

This study’s primary and secondary endpoints were 30-day and 1-year mortality, respectively. Cardiac death was defined as death resulting from arrhythmia, pump failure, or mechanical complications, including free-wall and ventricular septal ruptures. Death resulting from causes besides cardiac ones was considered a non-cardiac death.^[20]^ Regarding comparative analysis, continuous variables were compared using Student *t* test or the Wilcoxon rank-sum test, whereas categorical variables were compared using the *χ*^2^ or Fisher exact test.

The Kaplan–Meier method was used to estimate the incidence of clinical outcomes between the groups during follow-up, and the results were compared using the log-rank test. The weighted Kaplan–Meier method is used to control the censoring of survival data and can outperform the crude Kaplan–Meier method.^[21]^ Variables that could affect the outcomes, such as age, sex, Killip class, culprit-only PCI, thrombolysis in myocardial infarction (TIMI) major bleeding, acute kidney injury, multiorgan failure, and cardiopulmonary resuscitation, were used to calculate weights using a generalized linear regression model and compared using the weighted log-rank test. Finally, landmark analysis was performed at the 30-day mark, and the adjusted incidence was compared between the groups before and after 30 days.

A propensity score was calculated to assess the clinical outcomes through adjusted baseline clinical differences between the 2 groups.^[22]^ Multiple generalized linear regressions were performed to calculate the propensity score with ECMO support as the dependent variable. The variables that were significantly different between the groups were as follows: age, sex, Killip class, culprit-only PCI, for 30-days mortality and included TIMI major bleeding, acute kidney injury, multiorgan failure, and cardiopulmonary resuscitation for 1-year mortality.

Owing to the limited number of events that occurred in the study population, inverse probability of treatment weighting, calculated from the same variables used for propensity scoring, was used to estimate the average treatment effect of ECMO support and control for confounding factors.^[23]^

Comparisons of 30-day and 1-year mortality between the groups were based on Cox regression models, including unadjusted, propensity score-adjusted, and weighted models. Proportional hazard assumptions were checked using the log-log survival plot and Schoenfield residuals.

Simple and multiple Cox regression analyses were performed to identify the risk factors for 30-day mortality. A stepwise Cox regression model was created with bidirectional variable selection using the estimation of a function of the posterior probability of a model being true, under a certain Bayesian setup. In addition, another stepwise logistic regression model, which was created with bidirectional selection using Bayesian information criterion, was used to identify the risk factors for increased probability of requiring ECMO support at initial presentation. The following variables were included in the regression analysis: baseline characteristics, such as age, sex, comorbidities, hemodynamic data, and PCI characteristics, which showed significant associations in the simple logistic regression analysis (*P* < .1) with >90% data availability. Two-sided *P* values < .05 were considered statistically significant. Analyses were performed using the R software, version 3.1.1 (R Foundation for Statistical Computing, Vienna, Austria).

## 3. Results

**Figure 1.**
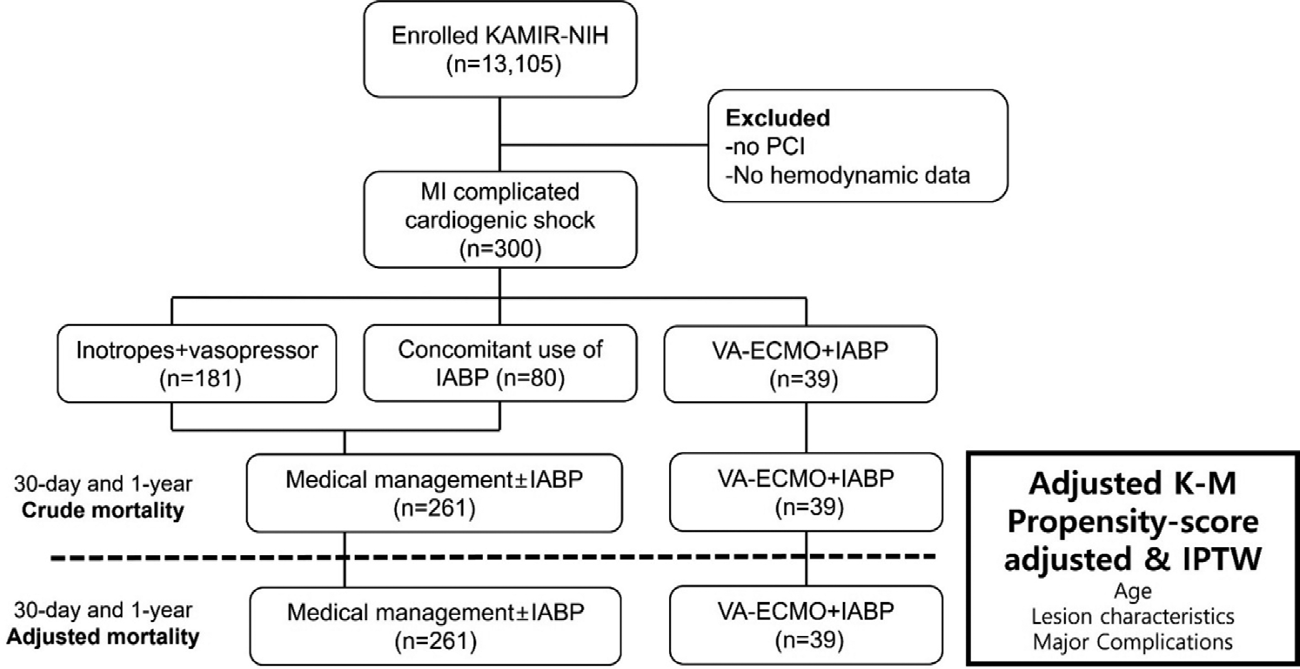
Flowchart of the study. IABP = intra-aortic balloon counterpulsation, IPTW = inverse probability of treatment weighting, KAMIR-NIH = the Korean Acute Myocardial Infarction Registry-National Institute of Health, K-M = Kaplan–Meier curve, MI = myocardial infarction, PCI = percutaneous coronary intervention, VA-ECMO = venoarterial extracorporeal membrane oxygenation.

During the study period, 13,105 patients were enrolled in the KAMIR-NIH registry at various study sites. After excluding patients whose coronary angiograms did not show coronary artery disease (n = 209) and those who did not undergo PCI (n = 128), underwent coronary artery bypass grafting (n = 37), were lost to follow-up (n = 127), had incomplete hemodynamic data (n = 345), and did not present with CS at initial presentation (n = 12,304), 300 patients with MI complicated by CS were included in this study. The included patients were categorized into VA-ECMO + IABP support (n = 39) and no VA-ECMO support (medical management ± IABP) (n = 261) groups (Fig. 1). Only 39 (13%) patients who were initially diagnosed with CS underwent ECMO in addition to IABP at the time of PCI. The remaining patients (n = 261, 87%) with CS were treated with only inotropes and vasopressors, and/or IABP (Fig. 1).

The baseline and procedural characteristics of the patients are shown in Table 1. There were some differences in the baseline characteristics of the 2 patient groups. Patients from the VA-ECMO + IABP support group were more likely to be young and men in contrast to the no VA-ECMO support group. Additionally, patients from the VA-ECMO + IABP support group had a higher Killip class than those from the no VA-ECMO support group (97.4% vs 70.9%; *P* = .006). The prevalence of cardiovascular risk factors in both groups was similar; however, history of angina or PCI was more common in the VA-ECMO + IABP support group than in the no VA-ECMO support group. The patients from the ECMO + IABP support group had higher initial serum hemoglobin, peak CK, and troponin levels than those from the no VA-ECMO support group. Hospital survivors from the VA-ECMO + IABP support group were prescribed aspirins, beta-blockers, angiotensin-converting enzyme inhibitors/angiotensin receptor blockers, and statins less frequently than those from the no VA-ECMO support group, which could have led to poor outcomes. Additionally, patients from the VA-ECMO + IABP support group showed multi-vessel involvement and left main artery disease, whereas patients from the no VA-ECMO support group showed single-vessel involvement with the right coronary artery as the culprit vessel. However, there were no differences in successful PCI, multi-vessel revascularization, and final post-procedural TIMI flow grade between the 2 groups. Low LV ejection fraction and high regional wall motion score were more common in the VA-ECMO + IABP support group than in the no VA-ECMO support group. However, there were no differences in volume and dimensional indexes between the 2 groups.

**Table 1:** Baseline characteristics of the study population, including angiographic and echocardiographic assessment data.

	No VA-ECMO N = 261 (87%)	VA-ECMO + IABP N = 39 (13%)	*P* value
Male, n (%)	177 (67.8)	34 (87.2)	.023
Age, median (SD)	67.31 (12.99)	62.10 (12.59)	.020
History of angina	34 (13.0)	3 (7.7)	.494
Systolic BP, mm Hg, median (SD)	54.08 (32.53)	47.82 (33.96)	.286
Diastolic BP, mm Hg, median (SD)	30.65 (22.61)	26.90 (24.42)	.371
Heat rate, per min, median (SD)	60.87 (38.36)	61.28 (41.87)	.954
Killip class, n (%)			
I	52 (19.9)	1 (2.6)	.006
II	13 (5.0)	0 (0.0)	
III	11 (4.2)	0 (0.0)	
IV	185 (70.9)	38 (97.4)	
Body mass index, kg/m^2^, median (SD)	23.29 (3.12)	24.14 (2.90)	.161
Hypertension, n (%)	149 (57.1)	21 (53.8)	.835
Diabetes, n (%)	99 (37.9)	13 (33.3)	.707
Dyslipidemia, n (%)	21 (8.0)	3 (7.7)	1.000
History of MI, n (%)	22 (8.4)	5 (12.8)	.369
History of angina, n (%)	19 (7.3)	7 (17.9)	.059
History of PCI, n (%)	26 (10.0)	9 (23.1)	.035
History of heart failure, n (%)	10 (3.8)	1 (2.6)	1.000
History of CVD, n (%)	22 (8.4)	3 (7.7)	1.000
Current smoker, n (%)	140 (53.6)	26 (66.7)	.176
Family history of CAD, n (%)	7 (2.7)	3 (8.1)	.119
WBC (×1000), median (SD)	13.42 (5.15)	14.22 (5.38)	.390
Serum Hb, median (SD)	12.67 (2.39)	13.73 (1.74)	.001
Serum glucose level, median (SD)	250.99 (123.01)	282.79 (128.39)	.159
Serum creatinine level, median (25th–75th quartile)	1.20 (0.90–1.50)	1.30 (0.95–1.70)	.144
Serum peak CK, median (25th–75th quartile)	670 (137–2515)	6632 (1383–12,938)	<.001
Serum peak troponin, median (25th–75th quartile)	31.51 (14.69–89.08)	153.48 (54.50–270.62)	<.001
High-density lipoprotein, median (SD)	38.31 (10.64)	34.35 (12.39)	.192
Low-density lipoprotein, median (SD)	96.81 (40.97)	81.68 (40.64)	.135
hsCRP, median (25th–75th quartile)	0.30 (0.10–1.76)	0.11 (0.04–0.96)	.152
BNP, median (25th–75th quartile)	105.00 (33.00–411.48)	535.00 (274.75–1058.00)	.656
HbA1c, median (25th–75th quartile)	6.10 (5.60–6.90)	6.05 (5.50–6.20)	.493
Discharge medications			
Aspirin, n (%)	256 (98.1)	36 (92.3)	.072
P2Y12inhibitor, n (%)			
Clopidogrel	169 (66.8)	25 (69.4)	.750
Ticagrelor	55 (21.7)	6 (16.7)	
Prasugrel	29 (11.5)	5 (13.9)	
Calcium channel blocker, n (%)	6 (2.3)	0 (0.0)	1.000
Beta-blocker, n (%)	157 (60.2)	5 (12.8)	<.001
ACEi and/or ARB, n (%)	147 (56.3)	7 (17.9)	<.001
Statin, n (%)	173 (66.3)	10 (25.6)	<.001
Hypoglycemic agent, n (%)	44 (100.0)	3 (75.0)	.083
PCI characteristics			
Access site, n (%)			
Radial	21 (8.0)	1 (2.6)	.330
Femoral	240 (92.0)	38 (97.4)	
GP IIb/IIIa inhibitor, n (%)	64 (24.5)	12 (30.8)	.523
Use of closure device, n (%)	44 (16.9)	2 (5.1)	.097
Target vessel, n (%)			
Left anterior descending	91 (34.9)	12 (30.8)	<.001
Left circumflex	21 (8.0)	5 (12.8)	
Right coronary	133 (51.0)	7 (17.9)	
Left main involve	16 (6.1)	15 (38.5)	
Lesion type of target vessel, n (%)			
Type A	1 (0.4)	0 (0.0)	.308
Type B1	19 (7.3)	2 (5.1)	
Type B2	99 (38.1)	21 (53.8)	
Type C	141 (54.2)	16 (41.0)	
Number of involved vessels, n (%)			
1-vessel disease	109 (41.8)	12 (30.8)	<.001
2-cessel disease	82 (31.4)	7 (17.9)	
3-vessel disease	45 (17.2)	4 (10.3)	
Left main involvement	25 (9.6)	16 (41.0)	
Pre-TIMI flow of target vessel, n (%)			
0	172 (66.2)	25 (64.1)	.969
1	22 (8.5)	4 (10.3)	
2	30 (11.5)	4 (10.3)	
3	36 (13.8)	6 (15.4)	
Treatment of target vessel, n (%)			
Balloon only	22 (8.4)	5 (12.8)	.369
Stent implantation	239 (91.6)	34 (87.2)	
Post-TIMI flow, n (%)			
1	6 (2.3)	2 (5.1)	.280
2	12 (4.6)	3 (7.7)	
3	243 (93.1)	34 (87.2)	
Type of index procedure, n (%)			
Culprit-only revascularization	214 (82.0)	32 (82.1)	1.000
Multi-vessel revascularization	47 (18.0)	7 (17.9)	
Staged PCI, n (%)	27 (10.3)	2 (5.1)	.396
Final diagnosis, n (%)			
NSTEMI	45 (17.3)	8 (20.5)	.792
STEMI	215 (82.7)	31 (79.5)	
Echocardiographic characteristics, median (SD)			
Left ventricle EF	48.47 (12.79)	32.85 (13.48)	<.001
Regional wall motion score index	1.52 (0.42)	1.81 (0.46)	.026
Left ventricle ESD	34.48 (8.88)	38.36 (8.69)	.160
Left ventricle EDD	49.15 (6.39)	48.36 (8.66)	.763
Left ventricle ESV	53.47 (36.63)	68.15 (31.13)	.211
Left ventricle EDV	100.33 (45.09)	104.79 (38.45)	.749

ACE = angiotensin-converting enzyme, ARB = angiotensin receptor blocker, BNP = B-type natriuretic peptide, BP = blood pressure, CAD = coronary artery disease, CK = creatinine kinase, CVD = cerebrovascular disease, EDD = end-diastolic dimension, EDV = end-diastolic volume, EF = ejection fraction, ESD = end-systolic dimension, ESV = end-systolic volume, Hb = hemoglobin, HbA1c = glycated hemoglobin, hsCRP = high- sensitivity C-reactive protein, IABP = intra-aortic balloon counterpulsation, LV = left ventricular, NSTEMI = non-ST segment elevation myocardial infarction, PCI = percutaneous coronary intervention, SD = standard deviation, STEMI = ST-segment elevation myocardial infarction, TIMI = thrombolysis in myocardial infarction, VA-ECMO = venoarterial-extracorporeal membrane oxygenation, WBC = white blood cell.

The crude incidence of major complications within 30 days in both groups is shown in Table 2. The incidence of TIMI major bleeding, ventricular fibrillation, acute kidney injury, multiorgan failure, and cardiopulmonary resuscitation was significantly higher in the VA-ECMO + IABP support group than in the no VA-ECMO support group. Severe bleeding^[24]^ (no data about the cause of bleeding) and multiorgan failure^[25]^ are well-known indicators of poor outcomes of CS.

**Table 2:** Comparison of major complications in both groups within 30 days.

	No VA-ECMO	VA-ECMO + IABP	*P* value
	N = 261 (87%)	N = 39 (13%)	
CVA, n (%)	0 (0.0)	1 (2.6)	.130
Brain hemorrhage, n (%)	1 (0.0)	2 (5.2)	.130
TIMI major bleeding, n (%)	18 (6.9)	15 (38.5)	<.001
TIMI minor bleeding, n (%)	19 (7.3)	7 (17.9)	.059
AV block, n (%)	43 (16.5)	4 (10.3)	.447
VT-controlled by antiarrhythmics, n (%)	14 (5.4)	4 (10.3)	.269
VT- controlled by shock, n (%)	57 (21.8)	14 (35.9)	.085
Ventricular fibrillation, n (%)	46 (17.6)	18 (46.2)	<.001
Atrial fibrillation, n (%)	43 (16.5)	6 (15.4)	1.000
Acute kidney injury, n (%)	8 (3.1)	6 (15.4)	.005
Multi-organ failure, n (%)	11 (4.2)	7 (17.9)	.004
Cardiopulmonary resuscitation, n (%)	140 (53.6)	37 (94.9)	<.001

AV = atrioventricular, CVA = cerebrovascular disease, IABP = intra-aortic balloon counterpulsation, TIMI = thrombolysis in myocardial infarction, VA-ECMO = venoarterial-extracorporeal membrane oxygenation, VT = ventricular tachycardia.

**Figure 2.**
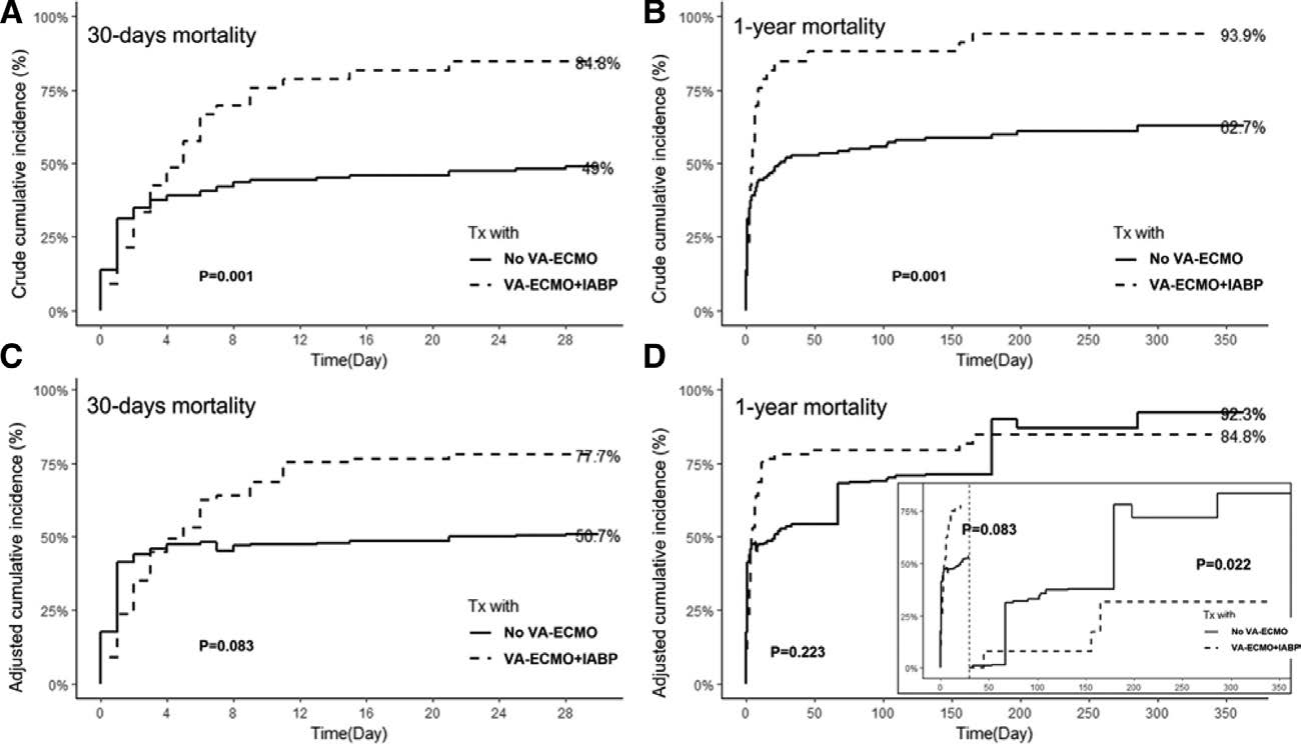
Crude cumulative incidence of (A) 30-day mortality and (B) 1-year mortality, and VA-ECMO + IABP versus no VA-ECMO support and adjusted incidence curve with weighted Kaplan–Meier methods for (C) 30-day and (D) 1-year mortality. Landmark analysis at the 30-day timepoint. IABP = intra-aortic balloon counterpulsation, Tx = treatment, VA-ECMO = venoarterial extracorporeal membrane oxygenation.

The cumulative incidence of 30-day mortality was 84.8% in the VA-ECMO + IABP support group and 49% in the no VA-ECMO support group (Fig. 2A). The difference in mortality between the groups was maintained at the 1-year follow-up (93.9% vs 62.7%; *P* = .001) (Fig. 2B). However, the adjusted cumulative incidence derived from the weighted Kaplan–Meier method by adjusting for confounding risk factors was different. The difference in 30-day mortality between the 2 groups decreased and lost its statistical significance (*P* value increased from 0.001 to 0.083) (Fig. 2C). After the extension of the follow-up period up to 1 year, there was no difference in mortality between the 2 groups (Fig. 2D). Landmark analysis at the 30-day timepoint showed that the adjusted incidence of mortality in the ECMO support group significantly decreased 30 days after the index hospitalization (Fig. 2D, picture-in-picture).

The propensity score-adjusted and weighted Cox regression models, which were constructed to adjust for possible confounding factors, showed similar results (Table 3). The crude risks of 30-day and 1-year mortality in the VA-ECMO + IABP support group were significantly higher than those in the no VA-ECMO support group (unadjusted hazard ratio [HR], 1.97 and 2.05; 95% CI, 1.26–3.09 and 1.34–3.13; *P* value, .003 and <.001; respectively). However, the propensity-adjusted and inverse probability of treatment weighting models showed no differences in 30-day (HR, 1.57; 95% CI, 0.92–2.77 *P* = .346 and HR, 1.44; 95% CI, 0.42–3.17; *P* = .452, respectively) and 1-year mortality (HR, 1.56; 95% CI, 0.95–2.56; *P* = .076 and HR, 1.33; 95% CI, 0.57–3.06; *P* = .51, respectively) between the groups.

**Table 3:** Outcomes of extracorporeal membrane oxygenation support.

	Unadjusted HR (95% CI)	*P* value	*PS-adjusted HR (95% CI)	*P* value	†IPTW HR (95% CI)	*P* value
30-d mortality	1.97 (1.26–3.09)	.003	1.57 (0.92–2.77)	.346	1.44 (0.42–3.17)	.452
1-yr mortality	2.05 (1.34–3.13)	<.001	1.56 (0.95–2.56)	.076	1.33 (0.57–3.06)	.510

CI = confidence interval, HR = hazard ratio, IPTW = inverse probability of treatment weighting, PS = propensity score.

*Adjusted PS model: propensity score-adjusted Cox regression model constructed using the following variables: age, sex, Killip class, culprit-only PCI, for 30-days mortality and included TIMI major bleeding, acute kidney injury, multiorgan failure, and cardiopulmonary resuscitation for 1-year mortality.

†IPTW model: weighted Cox model with calculated average weighted treatment effect constructed using the same variables used for the adjusted PS model.

**Figure 3.**
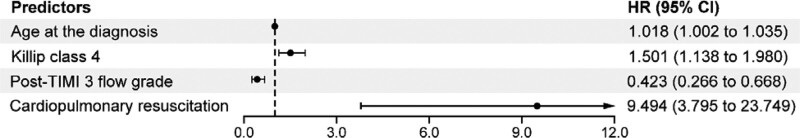
Independent predictors of 30-day mortality. CI = confidence interval, HR = hazard ratio, TIMI = thrombolysis in myocardial infarction.

A stepwise Cox regression analysis showed that old age (HR, 1.018; 95% CI, 1.002–1.035; *P* = .032), higher Killip class at initial presentation (HR, 1.501; 95% CI, 1.138–1.980; *P* = .067), low post-TIMI flow grade after PCI (HR, 0.423; 95% CI, 0.266–0.668; *P* ≤ 0.001), and cardiopulmonary resuscitation (HR, 9.494; 95% CI, 3.795–23.749; *P* < .001) were significantly associated with increased risk of 30-day mortality (Fig. 3). Another stepwise logistic regression analysis showed that patients with peak serum CK level at initial presentation were likely to receive ECMO support (odds ratio, 1.000093; 95% CI, 1.000183–1.000273; *P* < .001)

## 4. Discussion

The main finding of this study was that in the study population of patients with MI in CS, adjusted 30-day and 1-year mortality did not differ between the VA-ECMO + IABP support and no VA-ECMO support groups. However, the better survival benefit of VA-ECMO + IABP support 30 days after index hospitalization could be supposed. More patients from the VA-ECMO + IABP support group died between days 4 and 30 than from the no VA-ECMO support group. However, this finding was not statistically significant. Most patients with peak CK levels at initial presentation received early VA-ECMO + IABP support. Old age, high Killip class, non-recovery of TIMI flow grade after PCI, and cardiopulmonary resuscitation were independent predictors of 30-day mortality in patients with MI in CS.

CS is not a single disease entity caused by decreased cardiac output. The IABP-SHOCK study showed that the survival of patients with MI in CS after the initial 24 hours is not associated with the cardiac index.^[26]^ After initial shock occurred, clinical factors such as age, body mass index, lactate, anterior wall infarction, TIMI-3 flow after PCI, cardiopulmonary resuscitation time, and time from arrest to extracoporeal cardiopulmonary resiscitation may affect the clinical outcomes in MI-CS^[27]^; however, the main cause of mortality during shock is multiorgan dysfunction, which is triggered by severe systemic inflammation cascade,^[28]^ microvascular dysfunctions, and decreased perfusion to peripheral organs.^[25]^

To prevent multiorgan dysfunction, device-related complications should be minimized during shock.^[16]^ ECMO has several issues associated with an increased risk of access site bleeding and an increase in afterload, which may lead to increased end-diastolic pressure and LV volume. Severe bleeding complications during shock occur in 20 to 90% of CS cases and are associated with increased use of MCS. In addition, severe bleeding complications are not just related to the access site but to consumptive coagulopathy and acquired platelet dysfunction as well.^[24]^ A meta-analysis of 20 studies (1866 patients) indicated that the risk of major bleeding during ECMO support is 40.8% (26.8–56.6%). In addition, the study showed that the more severe the bleeding events, the higher the risk of 30-day mortality (bleeding: HR, 2.11; severe bleeding: HR, 2.80).^[29]^ This could explain the increased adjusted incidence of short-term mortality after 4 days among patients from the VA-ECMO + IABP support group, who were enrolled in a more severe shock state and had more 30-day complications, including bleeding, than those from the no VA-ECMO support group.

Increased afterload is another problem encountered while maintaining ECMO support. Various unloading techniques, such as concomitant use of IABP, impella, and left atrium or left pulmonary artery venting, have been described for the prevention of volume overloading to the left ventricle.^[30]^ IABP can also be a good option for an unloading technique during ECMO support. VA-ECMO could increase afterload, which would result in pulmonary edema.^[31]^ But ECMO support, in addition to IABP, have several beneficial effects: decreased myocardial oxygen demand and reduced pre-capillary wedge pressure in the pulmonary artery, which results in decreased hydrostatic pulmonary edema^[32]^; creates pulsatile perfusion flow, which could deactivate the coagulation pathway,^[33]^ protect from kidney injury,^[34]^ and improve lung perfusion^[35]^; increased hemodynamic profile in animal models, such as mean coronary artery flow, diastolic pressure time index, LV pressure-volume area, and tension time index.^[36]^ Recent study showed that ECMO, in addition to IABP support with proper management of device-related complications, may lead to improved clinical outcomes of CS in patients with MI (HR 0.30, 95% CI, 0.25–0.37), similar results as shown in the present study.^[37]^

The strength of this study was that long-term mortality included short-term mortality with VA-ECMO + IABP support, which we provided with adjusted incidence rates using various statistical methods. A recent systematic review showed that most studies regarding the clinical outcomes of ECMO support only reported crude incidence and indicated the harmful effects of ECMO during the early stage of MI-CS.^[38]^ Moreover, ECMO support, in addition to LV unloading, is crucial for improving the clinical outcomes of MI-CS,^[39]^ which our study reflected in a real-world clinical setting.

Several randomized controlled trials conducted to determine the efficacy of ECMO support for treating patients with MI-CS are ongoing. The ECLS-SHOCK (NCT03637205),^[40]^ EURO-SHOCK (NCT03813134),^[41]^ and ANCHOR (NCT04184635) trials are adequately powered to evaluate 30-day mortality as the primary endpoint. In addition, high-risk patients in CS are enrolled in these trials. Guidelines on the efficacy, proper management, and appropriate selection of ECMO support will be produced from these trials.

The present study had some limitations. First, this was a registry-based study and thus prone to selection bias. Although we used weighted Cox regression and adjusted Kaplan–Meier curves to control for such confounding factors, unidentified or residual confounders may have affected the study results. Second, the management of patients with MI in CS may vary depending on the volume of CS cases in an institution. Compared to hospitals with low volumes of CS cases, which may adopt a more defensive approach, hospitals with a large volume of CS cases have low mortality rates, even after adjusting for recent advances in the management of CS, including early revascularization.^[42]^ Third, experience in the initiation and management of ECMO support may have influenced the results. This includes different treatment durations of ECMO support among the study participants; therefore, we could not assess the correlation between treatment duration and clinical outcomes. Fourth, the lack of detailed information regarding ECMO support in special cases of cardiac arrest with ongoing cardiopulmonary resuscitation is another limitation. This is because ECMO cardiopulmonary resuscitation may be correlated with an increase in 30-day mortality by up to 13%.^[17]^ Additionally, the lack of details regarding the dosages of vasopressors and inotropes could have influenced the study results. There were also not enough data about the causes of the complications observed during the 30-day period. Finally, recent guidelines for the use of IABP in CS do not recommend it anymore; however, IABP is still used as a last-resort treatment for patients in severe CS to increase myocardial perfusion.^[43]^

In conclusion, this study demonstrated that patients with MI in CS treated with ECMO, in addition to IABP support, have comparable overall 30-day and 1-year risks of mortality compared to those not managed with ECMO. However, it appears that survivors after short-term ECMO support may have a better clinical outcome which should be considered hypothesis-generating.

## Acknowledgment

We would like to thank Editage (www.editage.com) for English language editing.

## Author contributions

**Conceptualization:** Jin Sup Park.

**Data curation:** Jeong Cheon Choe, Sun-Hack Lee, Jin Hee Ahn, Hye Won Lee, Jun-Hyok Oh, Jung Hyun Choi, Han Cheol Lee, Kwang Soo Cha, Myung Ho Jeong.

**Formal analysis:** Jin Sup Park.

**Funding acquisition:** Jin Sup Park.

**Investigation:** Jeong Cheon Choe, Sun-Hack Lee, Jin Hee Ahn, Hye Won Lee, Jun-Hyok Oh, Jung Hyun Choi, Han Cheol Lee, Kwang Soo Cha, Myung Ho Jeong.

**Methodology:** Jin Sup Park.

**Writing – review & editing:** Dominick J Angiolillo, Jin Sup Park.
